# Risk factors analysis for hyperuricemic nephropathy among CKD stages 3–4 patients: an epidemiological study of hyperuricemia in CKD stages 3–4 patients in Ningbo, China

**DOI:** 10.1080/0886022X.2018.1487859

**Published:** 2018-11-29

**Authors:** Yong-Yao Wu, Xiao-Hui Qiu, Yun Ye, Chao Gao, Fuquan Wu, Guihua Xia

**Affiliations:** a Beilun People’s Hospital & the Beilun Branch of the First Affiliated Hospital of School of Medicine, Zhejiang University, Ningbo, Zhejiang, China;; b Kidney Disease Center, Ningbo Medical Center (Li Huili Eastern Hospital), Ningbo, China;; c Department of Orthopedics, The First Affiliated Hospital of Soochow University, Orthopedic Institute, Medical College, Soochow University, Suzhou, Jiangsu, China

**Keywords:** CKD 3–4, hyperuricemic nephropathy, hUAT, serum uric acid, hyperuricemia

## Abstract

**Objective:** Uric acid (UA) is a risk marker of CKD and SUA level in CKD 3–4 patients closely correlates with hyperuricemic nephropathy (HN) morbidity. This study was designed to evaluate the risk factors for HN in CKD 3–4 patients.

**Methods:** The 461 CKD 3–4 patients were recruited and all patients were divided into three groups (24 h UUA normal, underexeret, and overproduct type groups) according to the 24 h UUA level after receiving low purine food for five days. Clinical and biochemical characteristics of CKD patients were collected for the logistic regression analysis. Correlation analysis of the mRNA relative expression level of hUAT and hURAT1 with serum UA (SUA) level also was evaluated.

**Results:** There were significant increases in characteristics including average age, waist-to-height ratio (WHR), SUA levels, HN ratio, TG/HDL ratio, body mass index (BMI), blood pressure (BP), uNgal/Cr. ratio, and uKim-1/Cr. ratio in overproduct type group in comparison with the other two groups. Logistic regression analysis showed SUA, CHO, uKim-1/Cr. ratio and uNgal/Cr. ratio were independent and multiple risk factors for HN. Moreover, hUAT and hURAT1 mRNA relative expression levels were significantly correlated with SUA level in the underexeret type CKD 3–4 patients.

**Conclusions:** These results showed SUA and other characteristics contributed to HN morbidity in CKD 3–4 patients.

## Introduction

Chronic kidney disease (CKD) is a clinical syndrome characterized by kidney function loss. Uric acid (UA) is a novel risk marker of CKD and might link mortality of CKD patients [1,2]. Hyperuricemia, defined as high serum UA (SUA), is prevalent in patients with CKD [1,3,4]. There are growing evidences that hyperuricemia is an independent risk factor for CKD or kidney fibrosis [5,6].

Hyperuricemic nephropathy (HN) or urate nephropathy, which is characterized by hyperuricemia, gout, tubulointerstitial nephritis, and kidney fibrosis, is a severe kidney disease mainly induced by excessive accumulation and deposition of uric acid salt in kidney [6,7]. Hyperuricemia is a poor prognostic factor of renal function in patients with IgA nephropathy [4,8]. Moreover, epidemiological surveys showed hyperuricemia also is the major etiological factor of gout and a marker of metabolic syndrome (MetS), which closely related to CKD, cardiovascular diseases, obesity and nephropathy [4,9]. Although some factors, signaling cascades, and genes had been reported to be associated with development of hyperuricemia and HN, the mechanism of hyperuricemia-induced HN, MetS, and CKD remains largely unknown so far.

Epidemiological surveys show sex, age, body mass index (BMI), blood pressure (BP), triglyceride (TG), renal failure, diabetes mellitus, waist circumference, waist-to-height ratio (WHR) are independent risk factors of hyperuricemia [10]. Moreover, dietary habit including fish consumption, alcohol consumption, smoking, is a recognized risk factor for elevated SUA levels and hyperuricemia. Ren et al. performed a 3-year follow-up study and confirmed that the consumption of raw or roasted fish, but not boiled or fried fish, was related to a high risk of hyperuricemia in Japanese adults [11]. The leading causes of CKD 3–4 include diabetes and hypertension [9,12]. In addition, the SUA level in CKD 3–4 patients closely correlates with HN morbidity [13–15].

Genetic factors including neutrophil gelatinase-associated lipocalin (Ngal) and kidney injury molecule-1 (Kim-1), both are renal early injury marker, enhanced in pediatric patients with hyperuricemia [3,16,17]. UA transporter 1 (URAT1) and urate transporter (UAT) which carry out urate efflux and exchange in the proximal tubule are elevated in hyperuricemia model [18–20]. The mutations, inhibition, or loss of function of hURAT1 has been reported to be associated with risk or occurrence of hypouricemia [21]. The inhibition of URAT1 and UAT related to decrement in SUA and hyperuricemia cure [18–20,22]. These reports showed URAT1 and UAT took crucial roles in hyperuricemia development. But it is uncertain whether URAT1 and UAT expression are risk factor for HN in CKD 3–4 patients or not.

This study was designed to evaluate the risk factors for HN in CKD 3–4 patients in Beilun, Ningbo, Zhejiang, China. Clinical and biochemical characteristics of CKD patients including sex ratio, age, BMI, WHR, SUA levels, HN ratio, TG/HDL ratio, SBP, BDP, uNgal/Cr. ratio, and uKim-1/Cr. ratio were detected and assessed. Logistic regression analysis and correlation analysis were performed to evaluate the contribution and correlation of those factors with HN risk in CKD 3–4 patients. This study would provide us with more information on the correlation of HN with CKD.

## Materials and methods

### Study cohort

The CKD 3–4 patients recruited in this study were from the Nephrology department, the Beilun People's Hospital, Ningbo, Zhejiang, China (Beilun Branch of the first Affiliated Hospital of Zhejiang University) during August 2014 and July 2016. Inclusion criteria for CDK stage 3–4 patients were: (1) aged 20- to 70-year old; (2) biopsy-proven primary glomerulopathy; (3) with new-diagnosed primary glomerulopathy without any drug therapy; (4) patients at CKD stages 3 to 4 determined according to the National Kidney Foundation criteria [23]. Moreover, the patients with one of diseases such as reflux nephropathy, obesity, HIV, and other infections, malignant cancers, autoimmune diseases, drug therapies (ACE-I/ARB, steroids, immunosuppressants, and etc.), and hereditary kidney diseases were excluded from this study. Accordingly, a total of 461 CKD 3−4 patients (male = 372, 80.7%; female = 89, 19.3%) were included. Then, the clinical data including blood pressure, body mass index (BMI), eGFR [24], perimeter of abdomen and hip, history of alcoholism and smoking, water and alcohol consumption, high purine food intake amount and frequency, and exercise were recorded.

### Ethics statement

This study was approved by the ethics review committee of the the first Affiliated Hospital of Zhejiang University, and was performed following the principles in the Helsinki Declaration II, with the written consents (*n* = 461) from all participants.

### Sample collection and patient groups

The 461 participants were fasting from 8:00 pm to the next day before renal biopsy at 8:00 am. The serum and urine samples were collected into sterile tubes without preservative and sent to the biochemical laboratory within 3 h at a cold condition (4 °C–6 °C). Automated urine analysis was performed on the iChem Velocity instrument according to described method [25], and other characteristics were measured using ELISA kits. Urine samples were collected from patients received low purine food for five days and urine uric acid (UUA) concentrations were determined. Hyperuricemic nephropathy (HN) patients were identified as CKD 3–4 patients with SUA concentration ≥420 μmol/L for males and ≥360 μmol/L for females [26]. After receiving low purine food for five days, CKD patients were grouped according to the UUA concentrations, as overproduct type (24 h UUA ≥3.6 mmol or ≥600 mg), underexeret type (24 h UUA 2.4–3.6 mmol or 400–600 mg), and normal type (24 h UUA 1.2–2.4 mmol or 200–400 mg). Biopsy samples of renal tubular epithelial cells from three groups were snapped in liquid nitrogen and stored at −80 °C for preparation of mRNA analysis and biochemical measurements. Hyperuricemia was diagnosed as SUA levels ≥6 mg/dL in women and ≥7 mg/dL in men.

### Biochemical measurements

Quantitative measurement of serum characteristics including SUA, XOR, C-reactive protein (CRP), and urine features such as UUA, creatinine (Cr), Ngal, and Kim-1 in morning serum and urine samples fasting for 12 h were measured using commercial enzymatic kits according to the manufacturers’ protocols. Serum lipid profile (TG, LDL, CHO) were determined by the enzymatic method using the Hitachi 912 chemistry analyzer.

### Quantitative real time PCR for hUAT and hURAT1 mRNA expression

The expressions of hUAT and hURAT1 mRNA in biopsy samples were detected using Quantitative real time PCR (qRT-PCR). Total tissue RNA was isolated using the Trizol reagent (Invitrogen Corp., Carlsbad, CA) for synthesis of the first strand cDNA, which were then used for the amplification DNA templates. ABI7500 Real-Time PCR system (Applied Biosystems) was employed for qRT-PCR analysis and reactions were conducted as follows: 95 °C 60 s, followed by 40 cycles of 95 °C 30 s, 60 °C 30 s, and finally at 70 °C for 30 s. All reactions were run in triplicates. Relative expression levels of hUAT and hURAT1 mRNAs were detected using 2^- △△Ct^ method, with normalization to GAPDH expression.

### Statistical analysis

IBM statistics (version 17.0) or GraphPad Prism 6 (Graphpad Software, San Diego, CA) was used for statistical analyses. Continuous variables were reported as means ± SEMs while categorical variables were expressed as proportions. All analyses were performed with adjustment for age and gender. Differences between two groups were analyzed using *χ*^2^ test or *t* test, or one-way ANOVA test for categorical variables or continuous variables followed the normal distribution, respectively. Differences among three groups were detected using Fisher's Test. Spearman’s correlation test was used for correlation analysis between SUA and hUAT or URAT1 mRNA expression levels. Prior to analysis, concentrations of Ngal and was adjusted to Cr, as Ngal/cr. Multiple linear regression models were created to determine the risk factors for CKD. For all analysis, *p* < .05 was regarded as significant.

## Results

### Biochemical characteristics of CKD patients

The primary clinical and biochemical characteristics of these 461 CKD 3–4 participants are statistically analyzed and listed in Table 1. A total of 372 male (80.7%) and 89 female (19.3%, *p* < .0001) CKD3–4 patients were included in this present study. Age of males was younger than that of females (42.4 ± 8.3 vs. 54.4 ± 7.5, respectively). Primary SUA and UUA of males was higher than that of females (SUA 432.7 ± 67.3 μmol/L vs. 382.4 ± 48.1 μmol/L, respectively; 4.7 ± 2.2 mmol/L vs. 3.9 ± 1.3 mmol/L, respectively). The HN morbidity of males was higher than that of females [85.5%, (318/372) vs. 59.6%, (53/89), respectively].

**Table 1.** The clinical and biochemical characteristics of CKD patients in this study and the differences among groups.

Parameters	Groups			*p* value
	Normal type	Underexeret type	Overproduct type	
Patients (*n*, %)	193 (41.87%)	164 (35.57%)	104 (22.56%)	<.0001
Age (years)	45.9 ± 7.3	48.1 ± 10.0	56.9 ± 9.2	<.0001
BMI	23.1 ± 3.3	24.1 ± 4.2	25.6 ± 3.4	=.0284
WHR	0.9 ± 0.3	1.0 ± 0.4	1.1 ± 0.3	=.0320
Roasted fish consumption (g/d)	11.9 ± 6.6	18.3 ± 6.8	35.9 ± 11.9	<.0001
Alcohol consumption (mL/d)	20.6 ± 7.1	33.5 ± 10.5	42.9 ± 13.9	<.0001
Smoking (times/d, mean)	8.8 ± 5.3	12.4 ± 5.7	16.8 ± 10.4	=.0763
Water consumption (L/d)	1.4 ± 0.7	1.1 ± 0.4	0.9 ± 0.4	=.0219
Primary SUA (μmol/L)	353.9 ± 68.1	387.8 ± 62.4	398.8 ± 63.4	=.0274
Primary UUA (mmol/L)	4.0 ± 0.6	4.3 ± 0.7	4.7 ± 0.8	=.2976
HN (%, *n*)	68.4% (132/193)	87.6% (147/164)	88.6% (92/104)	<.0001
p.5 d SUA (μmol/L)	290.6 ± 67.7	315.1 ± 61.8	350.5 ± 57.9	=.0071
p.5 d UUA (mmol/L)	1.9 ± 0.4	3.2 ± 0.3	4.5 ± 0.8	<.0001
TG/HDL ratio	3.1 ± 1.1	3.4 ± 0.9	3.9 ± 0.5	=.0027
CHO (mg/dL)	4.7 ± 0.8	4.9 ± 1.0	5.3 ± 1.4	=.0827
SBP (mmHg)	134.8 ± 13.2	140.7 ± 11.0	158.7 ± 15.0	<.0001
DBP (mmHg)	95.1 ± 7.2	99.9 ± 8.8	101.5 ± 6.9	=.0165
uNgal/Cr. ratio (ng/mL Cr.)	4.5 ± 0.8	5.1 ± 2.2	7.3 ± 2.3	<.0001
uKim-1/Cr. ratio (ng/mL, Cr.)	1.5 ± 0.8	3.6 ± 0.9	7.3 ± 2.3	<.0001
eGFR (mL/min/1.73 m^2^)	109.6 ± 19.6	102.5 ± 23.9	98.1 ± 10.6	=.0125
XOD (U/L)	3.5 ± 2.6	4.7 ± 4.1	8.8 ± 3.7	<.0001
CRP (mg/L)	26.8 ± 4.1	33.4 ± 7.8	40.4 ± 7.9	<.0001

From up to down: BMI: body mass index; WHR: waist hip ratio; SUA: serum uric acid; UUA: urine uric acid; HUA: hyperuricemia; TG: triglycerides; HDL: high-density lipoprotein; HN: hyperuricemic nephropathy; CHO: cholesterol; SBP: systolic blood pressure; DBP: diastolic blood pressure; uNGAL: urine neutrophil gelatinase-associated lipocalin; uKim-1: urine kidney molecule-1; Cr: creatinine; eGFR: estimated glomerular filtration rate; XOD: xanthine oxidase; CRP: C-reactive protein. p(0).5 d: indicates post five days.

The 461 CKD patients were grouped into three groups according to the 24 h UUA concentrations after receiving low purine food for five days. Patients showed normal 24 h UUA level (1.2–2.4 mmol or 200–400 mg/d), underexeret 24 h UUA level (2.4–3.6 mmol or 400–600 mg/d), and overproduct 24 h UUA level (≥3.6 mmol or ≥600 mg/d) after receiving low purine food for five days were grouped into the corresponding group [Table 1, *n* = 193 (41.87%), 164 (35.57%), and 104 (22.56%), respectively (*p* < .0001); UUA levels after 5 d = 1.9 ± 0.4 mmol/L, 3.2 ± 0.3 mmol/L, and 4.5 ± 0.8 mmol/L, respectively (*p* < .0001)].

### Differences analysis for characteristics of CKD patients among three groups

Statistical analyses for the primary clinical and biochemical characteristics of these 461 participants were performed among the three groups (Table 1). Analysis showed that there were differences in some characteristics among three groups. Patients of overproduct type showed higher age, BMI, WHR, roasted fish consumption, alcohol consumption, primary SUA levels, HN ratio, SUA, and UUA levels after 5 d low purine food, TG/HDL ratio, SBP, BDP, uNgal/Cr. ratio, uKim-1/Cr. ratio, XOR and CRP concentrations than those of the other groups (*p* < .05). On contrast, patients of overproduct type showed lower water consumption (1.4 ± 0.7 L/d, 1.1 ± 0.4 L/d, vs. 0.9 ± 0.4 L/d, respectively, *p* = .0219) and eGFR (109.6 ± 19.6 mL/min/1.73 m^2^, 102.5 ± 23.9 mL/min/1.73 m^2^, vs. 98.1 ± 10.6 mL/min/1.73 m^2^, respectively, *p* = .0125). Moreover, no differences were observed in smoking (*p* = .0763), primary UUA levels (*p* = .2976), and serum CHO concentrations (*p* = .0827) among three groups, although there were uptrends (Table 1).

### Multiple regression analysis of hURAT1

The factors had significant differences among groups in Table 1 were used as explanatory variables to create the multiple regression models, with elimination of some significant variables. Multiple regression analysis procedure created one model in Table 2. Table 2 shows the remaining four factors (SUA, CHO, SBP, uNgal/Cr. ratio) accounted for about 47% variations in hURAT1 mRNA relative expression level (*R* = 0.679, *p* < .001, Table 2).

**Table 2.** Multiple linear regression analysis of the URAT1 mRNA relative expression level.

Variables	Coefficient	OR (95% CI)	*p* value
SUA (μmol/L)	1.125	3.435 (1.762−5.493)	<.001
CHO (mg/dL)	0.522	1.673 (1.079−2.681)	.012
SBP (mmHg)	0.518	1.520 (1.127−2.798)	.011
uNgal/Cr. ratio (ng/mL Cr.)	0.335	1.803 (1.592−2.985)	.030

From up to down: SUA: serum uric acid; CHO: cholesterol; SBP: systolic blood pressure; uNGAL: urine neutrophil gelatinase-associated lipocalin; Cr.: creatinine.

### Correlation analysis of hUAT and hURAT1 with SUA

**Figure 1.**
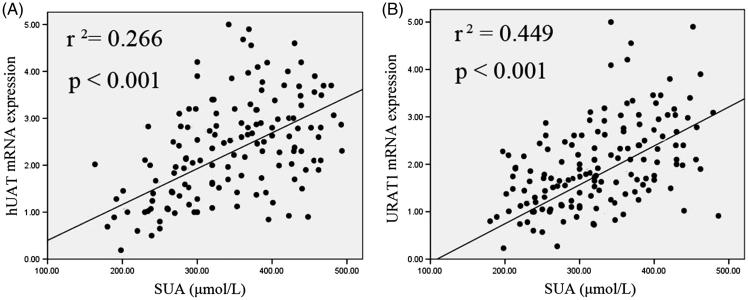
Correlation analysis for hUAT and hURAT1 mRNA relative expression levels with SUA concentrations in underexeret CKD patients.

We detected the mRNA expression of hUAT and hURAT1 mRNA in biopsy samples of renal tubular epithelial cells from underexeret CKD patients (*n* = 164) using qRT-PCR. We then performed the correlation analysis for hUAT and hURAT1 mRNA relative expression levels and SUA concentrations in underexeret CKD patients and confirmed the correlation of hUAT and hURAT1 relative mRNA expression level and SUA content (Figure 1).

## Discussion

This epidemiological study examined the incidence of HN among CKD 3–4 patients, and examined the risk factors for HUA. CDK 3–4 patients were grouped into three groups (normal, underexeret, and overproduct type groups) according to the 24 h UUA level after receiving low purine food for five days. We analyzed there were differences in clinical and basal characteristics among three groups, and evaluated that SUA level, roasted fish consumption, SBP, uNgal/Cr. Ratio, and uKim-1/Cr. Ratio are the risk factors for HN among CKD 3–4 patients. Moreover, the correlation analysis for hUAT and hURAT1 mRNA relative expression level showed the positive correlation of them with SUA content. These demonstrated that both hUAT and hURAT1 might be potential risk factors for the development of HN among CKD 3–4.

Our study showed the HN incidence, male sex ratio, age, BMI, WHR, roasted fish consumption, alcohol consumption, SUA levels, SBP, uNgal/Cr. ratio, uKim-1/Cr. ratio were different in three groups with different UA metabolism. Ren et al. showed that roasted and raw fish consumption contributed to hyperuricemia [11]. Evidence have shown factors such as sex, alcohol consumption, and age are risk factors of CKD and hyperuricemia [1,27]. Kim-1 and Ngal are sensitive marker of early kidney injury, and expression of them could be enhanced in hyperuricemia [3,16,17]. We confirmed that roasted fish consumption, alcohol consumption, sex, and CHO level, uKim-1/Cr. ratio, and uNgal/Cr. ratio were risk factors for HN in CKD 3–4 in this present study. These demonstrated that those factors might be associated and contributed to the accordance of HN from CKD 3–4.

Reports show hyperuricemia is also associated with cardiovascular diseases, metabolic syndrome, renal disease, and increased mortality [8]. Hyperuricemia might be a risk factor of developing contrast-induced nephropathy, renal insufficiency, or closely associated with CKD [5,28]. In this present study, we confirmed that the HN morbidity in CKD 3–4 was increased among three groups, accompanied with the eGFR reduction and SBP increment (Table 2). This is in accordance with the fact of hyperuricemia is largely prevalent in patients with CKD [1]. In addition, in this present study we confirmed that the HN morbidity in overproduct UUA type and underexeret UUA type of CKD 3–4 patients was over 87.6% and 88.6%, respectively, versus 68.4% in normal UUA type patients (*p* < .0001). These suggested that the HN morbidity correlated with UA level, renal insufficiency, or UA metabolism in CKD 3–4 patients in Beilun, Ningbo.

URAT1 exchanges proximal tubule lumen urate, and urate secretion to the lumen is carried out by UAT [29,30]. In addition, UAT supports urate reabsorption [30]. Some studies had shown that the expression of URAT1 associated with UA levels in hyperuricemia rats [19,20,22]. Xu et al. had reported that the oral administration of *Rhizoma Smilacis Glabrae* extract to hyperuricemic rats could reduce expression of URAT1 mRNA and UA levels [19]. Chen et al. confirmed that the elevated SUA level and enhanced expression of URAT1 mRNA and protein in chronic hyperuricemia rats could be reduced by total saponin of *Dioscorea* [22]. Sun et al. confirmed that Compound Tufuling Granules could reduce the expression of UAT and SUA level in hyperuricemia rats [18]. Other reports showed the therapeutic effect of traditional Chinese medicine or agents on hyperuricemia and inhibition of URAT1 and UAT demonstrated URAT1 and UAT took roles in hyperuricemia development. In this present study, we concluded that the expression of hURAT1 and hUAT mRNA correlated with SUA. These suggested that the hURAT1 and hUAT mRNA level might be used as a risk factor for HN.

## Conclusion

In conclusion, we found HN incidence, male sex ratio, age, BMI, WHR, roasted fish consumption, alcohol consumption, SUA levels, SBP, uNgal/Cr. ratio, and uKim-1/Cr. ratio were different in three groups with different UA metabolism, and all increased with UA dysfunction. Moreover, we demonstrated that the occurrence of HN in CKD stages 3–4 cohort was associated with sex, roasted fish consumption, alcohol consumption, CHO level, SBP, uNgal/Cr. ratio, and uKim-1/Cr. ratio. Further interventional or experimental studies would be done to confirm the causality and mechanism of these relationships.
